# Effects and mechanism of myeloperoxidase on microglia in the early stage of intracerebral hemorrhage

**DOI:** 10.3389/fnins.2022.1046244

**Published:** 2022-12-09

**Authors:** Wei Zuo, Yunchang Wang, Jiali Sun, Yinian Zhang

**Affiliations:** ^1^Department of Neuro-Oncological Surgery, Neurosurgery Center, Zhujiang Hospital of Southern Medical University, Guangzhou, China; ^2^Xiangya Hospital, Central Southern University, Changsha, China; ^3^Department of Neurosurgery, Lanzhou University Second Hospital, Lanzhou, China; ^4^College of Life Sciences, Central Southern University, Changsha, China

**Keywords:** myeloperoxidase, intracerebral hemorrhage, microglia, activation, inhibition, neurobehavior

## Abstract

**Objectives:**

(1) To clarify the dynamic relationship between the expression of myeloperoxidase (MPO) and microglial activation of intracerebral hemorrhage (ICH), (2) to explore the effect of inhibition of MPO on microglial activation, and (3) to observe the improvement in the neurobehavior of mice with inhibition of MPO.

**Methods:**

C57 BL/6 mice and CX3CR1 + /GFP mice were used to establish a phosphate-buffered saline (PBS) group, an ICH group, and a 4-aminobenzoic acid hydrazide (ABAH) group. Longa score, open field locomotion, hind-limb clasping test, immunohistochemistry, immunofluorescence, blood routine detection, and flow cytometry were used.

**Results:**

The neurobehavior of the mice was significantly impaired following ICH (*P* < 0.01); the expression of MPO was significantly increased following ICH, and reached a peak value at 6 h post-injury (*P* < 0.001). Moreover, the microglial activation increased significantly following ICH, and reached a peak level at 24 h post-injury (*P* < 0.01). Following inhibition of MPO, the activation of microglia in the ICH group decreased significantly (*P* < 0.001). Moreover, the neurobehavior of the ICH group was significantly improved with MPO inhibition (*P* < 0.05).

**Conclusion:**

MPO may be an upstream molecule activated by microglia and following inhibition of MPO can improve secondary injury resulting from ICH.

## Introduction

Intracerebral hemorrhage (ICH) accounts for 10–15% of all strokes and is characterized by high lethality and disability. ICH damage to the brain consists of either primary hematoma compression effects or secondary hematoma breakdown products, both of which cause damage to the brain parenchyma. The main factor leading to poor prognosis of ICH is secondary brain injury. The mechanisms that cause secondary injury to ICH are mainly inflammatory response, local active oxygen free radical release, and apoptosis around hematomas (Xi et al., 1999; Cherubini et al., 2000; Wang et al., 2007). ICH results in neuronal cell death and the release of factors such as damage-associated molecular patterns (DAMPs) that induce localized inflammation in the injured brain region. And such focal brain inflammation aggravates secondary brain injury by exacerbating blood-brain barrier breakdown, microvascular failure, brain edema, oxidative stress, and by directly inducing neuronal cell death (Shi et al., 2019). Therefore, the inflammatory response is currently considered to be the key factor causing secondary brain injury following ICH (Wang et al., 2007). Inflammatory responses include microglial activation, leucocyte infiltration, enzyme activation, and release of numerous mediators of injury, such as hemoglobin, iron, reactive oxygen and nitrogen species (Garcia et al., 1994).

Following ICH, microglial cells, as important neuroinflammatory effector cells, are the first non-neuronal cells to generate an immune response to acute brain injury (Walentynowicz et al., 2018). *In vitro*, activated microglia are polarized, showing two polarizing phenotypes, “classically activated” proinflammatory (M1) or “alternatively activated” anti-inflammatory (M2) cells (Xiong et al., 2016). At present, it is believed that when ICH occurs, microglia are activated and the phenotype undergoes short-term dynamic changes (Wang et al., 2013; Lee et al., 2016; Liu et al., 2021). The expression of the M1 microglia in the early stage of ICH is much higher than that of M2 microglia, showing a global pro-inflammatory state (Lan et al., 2017). M1 macrophages are neurotoxic, and M2 macrophages promote a regenerative growth response in adult sensory axons (Kigerl et al., 2009). M1-type microglia are produced and release a series of inflammatory mediators and biologically active factors, such as TNF-α, IL-1β, IL-6, IL-8, etc., lead to inflammation following ICH, and cause further secondary damage. Therefore, microglia plays a key role in the initial inflammatory response following ICH. Supportive treatment is currently the main therapy for ICH (Thabet et al., 2017). A recent study has shown that use of pinocembrin (molecular formula: C_15_H_12_O_4_) to inhibit M1 microglia can protect brain tissue following ICH (Lan et al., 2017). However, the specific mechanism of microglial activation and polarization in ICH is still unclear.

Myeloperoxidase (MPO) is a leukocyte enzyme secreted by activated neutrophils, monocytes, and macrophages, and it possesses peroxidase activity (Arnhold and Flemmig, 2010). Experiments have shown that, in addition to being abundant in neutrophils, MPO is also expressed in other myeloid cells, such as microglia (Gray et al., 2008). In a mouse model of ICH, bleeding causes upregulation of inflammatory factors in the brain, a large number of blood myeloid cells are recruited into the brain, and MPO is synthesized and released to evacuate hematomas. On the other hand, the inflammatory network regulated by MPO may trigger subsequent injury. Therefore, MPO is considered to be a biomarker for the diagnosis and prognosis of ICH (Wang et al., 2007). Studies have shown that the classic MPO inhibitor, 4-aminobenzoic acid hydrazide (ABAH), can inhibit MPO activity, increase the proliferation of stroke neurons, and improve neurogenesis (Forghani et al., 2015; Kim et al., 2016). Stefanova et al. (2012) found that inhibition of MPO in PLP-a-SYN mice can inhibit microglial cell activation and, at the same time as MPO peaks at 6 h, M1 microglial cells are also activated. From the results of previous studies, it is not difficult to speculate that there may be some connection between MPO and microglia in ICH. Therefore, we hypothesized that the key target cells regulated by MPO in ICH are microglial cells. The activating effect of MPO on microglial cells was verified by experiments, and the effects and mechanism of MPO on microglial cells in the early stage of ICH were investigated. This could provide an endogenous treatment strategy for clinically difficult ICH cases.

## Materials and methods

### Experimental animals

The C57BL/6 mice and CX3CR1 + /GFP mice used in this experiment (had the same genetic background as C57BL/6 mice, including immune cells of myeloid origin, including microglia in the central nervous system; expression of the green fluorescent protein (GFP) gene was composed and provided by the Medical Laboratory Animal Center of Lanzhou University) were kept in a quiet environment, freely fed and watered, keep the living environment of mice for 12 h each day and night. Of these, 37 C57BL/6 mice were male, 8 weeks old, weighing 20 ± 2 g; 21 CX3CR1 + /GFP mice were all male, 8 weeks old, weighing 20 ± 2 g. All animal experiments were approved by the Medical Laboratory Animal Ethics Committee of Lanzhou University Second Hospital. All animals were euthanized in accordance with ethical animal laboratory practice.

### Establishment of intracerebral hemorrhage model

All experimental mice were placed in the experimental environment for at least 2 weeks prior to experimentation in order to adapt the mice to the environment, allowing the mice free access to food and water. The animals were anesthetized by intraperitoneal injection with a 1% sodium pentobarbital solution (50 mg/kg), and then venous blood from the mice was added to an EP tube soaked with heparin using the tail-capped blood collection method. The temperature of the mice was maintained at 37 °C using a thermostatic blanket. The individual mice were placed in a stereotactic frame, the skin of the head was cut, and the scalp was wiped with a hydrogen peroxide solution so that the skull was fully exposed, and a skull hole (approximately 1 mm in diameter) was drilled on the right side of the sagittal line. A 26-gauge needle was inserted into the striatum on the right side (coordinates: *X* = −2.0 mm; *Y* = −0.5 mm; *Z* = −3.5 mm), and a micro-infusion pump was used, the ICH mice were perfused with 10 μL of autologous blood, and the control mice were perfused with ice-cold phosphate-buffered saline (PBS; pH 7.4). The needle was removed, the drilled hole was filled with bone wax, and the skin incision was sutured with a No. 4 suture.

### Behavioral experiments

**Longa score:** A score of 2–3 indicates that the model was successfully created.

0 points: normal, no neurological deficits.
1 point: left forelimb cannot fully extend, mild neurological deficit.
2 points: when walking, mice looped to the left, with moderate neurological deficits.
3 points: when walking, mice collapsed to the left, with severe neurological deficits.
4 points: mice cannot walk on their own, losing consciousness.

**Open field locomotion:** We used opaque plastic to make a cube box of dimensions 50 cm × 50 cm × 50 cm. The bottom of the box and the inner walls were white. The mouse was placed at a specific corner facing the central area and timing was commenced. The mouse’s activities within 15 min were automatically recorded. The open field in the video was divided into a central area and a surrounding area by SMART v3.0. The central area accounted for 25% of the total area, and the mouse’s movements were tracked simultaneously. We observed the exercise distance, exercise speed, rest time, and the time the mouse spent in the central area in order to evaluate the exercise ability of the mouse.

**Hind-limb clasping test:** The tail suspension of the mouse was lifted, the movements of the hind limbs of the mouse within 15 s were recorded, and the hind-limb clasping and muscle strength were observed.

### Immunohistochemical and immunofluorescence labeling

We first divided the mice into an ICH group and a PBS group and observed iba1 antibody cells and MPO staining at 6, 12, and 24 h. Six C57 mice were used in each group. We then used the CX3CR1 + /GFP mice for MPO immunofluorescence with six mice per group. We used 40 μm-thick brain slices for immunohistochemistry. For iba1 staining, we used the ABC method. The antibodies were Rabbit anti iba1 (Solarbio Life Sciences, Beijing, China, 1:600), Goat anti Rabbit (Solarbio, 1:300), and streptavidin–horseradish peroxidase conjugate (STR-HRP). For MPO fluorescence staining, we used Rabbit anti MPO (Solarbio, 1:600) and Goat anti Rabbit (Solarbio, 1:300). Following each antibody staining, the brain slices were washed three times with PBST (PBS + Triton). Images of the immunohistochemically labeled sections and the fluorescently immunolabeled sections were obtained using an Olympus CKX41 fluorescence inverted microscope (Olympus, Tokyo, Japan). The images were captured in the FITC and TRITC channels using Cell-P imaging software (Olympus). When the two images were merged, the cells appeared yellow.

### Blood routine detection

Before perfusion and taking of the brain slices, 1 ml of blood was collected using the orbital sinus blood collection method in an EP tube soaked with heparin. The EP tube was placed in a routine blood testing machine, and the results were recorded automatically.

### Flow cytometry

The brain tissue was placed in pre-chilled flow cytometry buffer [1% bovine serum albumin (BSA), 1 mM EDTA, pH 7.4, 0.1 M PBS, 50 U/ml DNAseI], the striatal area was gently shaken three times, and the tissue was broken up and passed twice through a 70 μm nylon filter in order to generate a single cell suspension, and then the cells were centrifuged at 4 °C, 1,200 rev/min, the supernatant was discarded, flow buffer was added, and the cells washed with 1 × flow cytobuffer, at 4 °C, 1,200 rev/min. After discarding the supernatant and transferring the suspension to a flow tube, flow cytometry was performed. The FITC channel was used for screening. The results were analyzed using FlowJo software.^1^

## Statistical analysis

All data were expressed as the mean ± standard error on the mean (SEM). Comparison between two groups was performed using the unpaired Student’s *t*-test. Comparison between multiple groups was performed using one-way analysis of variance (ANOVA) following the *post-hoc* test. *P* < 0.05 was taken to be statistically significant. Prism 8 statistical graphing software^2^ was employed, and FlowJo software was used for flow cytometry analysis.

## Results

### Inhibition of myeloperoxidase activity promotes recovery of motor function following intracerebral hemorrhage

**FIGURE 1:**
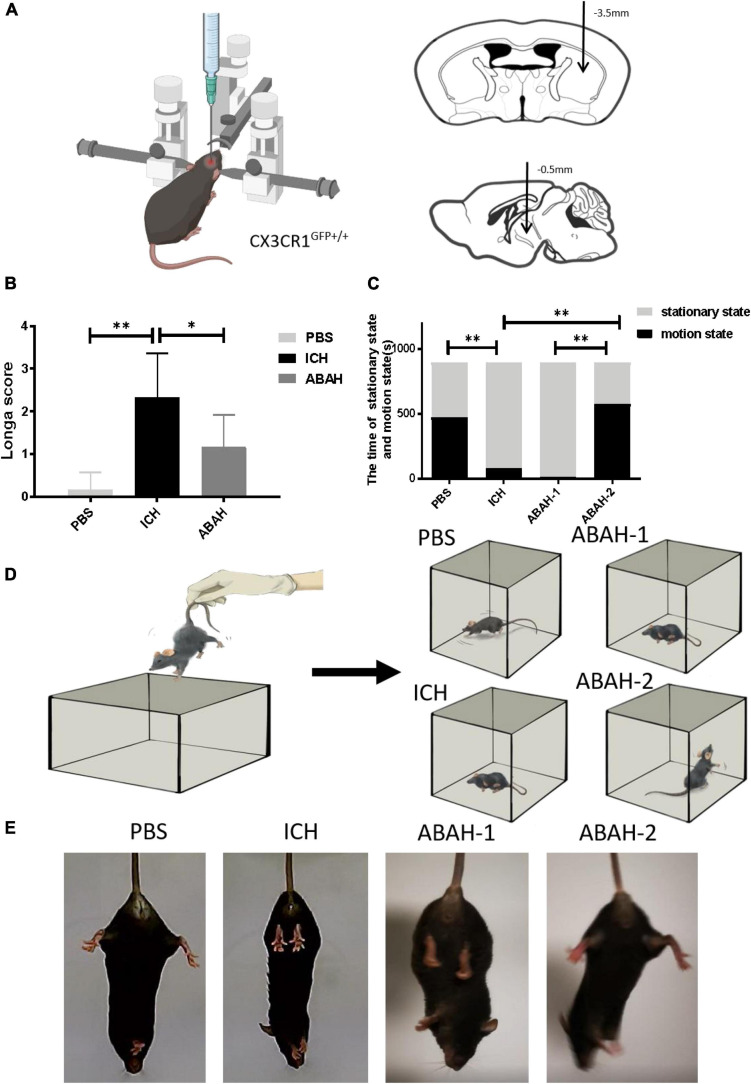
Inhibition of myeloperoxidase (MPO) activity promotes recovery of motor function following intracerebral hemorrhaging (ICH). **(A)** Establishment of intracerebral hemorrhage model; **(B,C)** Longa scores of PBS, ICH, and ABAH groups (*P < 0.05, **P < 0.01, *n* = 6 in each group); open field locomotion in the PBS, ICH, and first and second ABAH treatment groups. The times of movement and rest of the mice in the open field were recorded. The total time was 900 s (*P < 0.05, **P < 0.01, *n* = 6 in each group); **(D)** open field mode diagram; **(E)** hind-limb clasping test in the PBS, ICH, and first and second ABAH treatment groups. The degrees of hind-limb opening and closing in mice were observed.

In order to explore whether the activity of MPO affects the recovery of motor function following ICH, we injected ABAH into the striatum (CPu) of mice in order to inhibit the activity of MPO (Figure 1A). We used the Longa score, the open field test, and the hind-limb clasping test to comprehensively evaluate the motor behavior ability of the control, ICH, and ABAH intervention groups, respectively, (ABAH-1/ABAH-2). The Longa score showed that, compared with the PBS group, the neurological deficits in the ICH group were more obvious, and the neurological behavior of the ABAH group was improved compared with that of the ICH group (Figure 1B). In order to confirm that ABAH intervention improved neurological function following ICH, we used the open field test to evaluate the motor ability of experimental animals (Figures 1C,D). The PBS, ICH, and ABAH-2 groups were tested at 24 h, and the ABAH-1 group was tested at 6 h, because the ABAH-1 group was given the first dose of ABAH before the model was established, and the ABAH-2 group was given its dose after the behavior of the ABAH-1 group was tested. The results showed that, compared with the PBS group, the exercise time of the ICH group was significantly reduced, while the difference between the ABAH-1 group and the ICH group was not statistically significant, but the exercise state of the ABAH-2 group was significantly improved (Figure 1C). In the hind-limb clasping experiment, we found that, compared with the PBS group, the ICH and ABAH-1 groups had weaker hind-limb clasping ability and more obvious symptoms of paralysis. The hind-limb clasping ability of the ABAH-2 group was significantly improved (Figure 1E).

In summary, we found that use of the MPO activity inhibitor ABAH significantly improved motor ability following ICH.

### Intracerebral hemorrhage can induce changes in the number and proportion of inflammatory cells in peripheral blood

**FIGURE 2:**
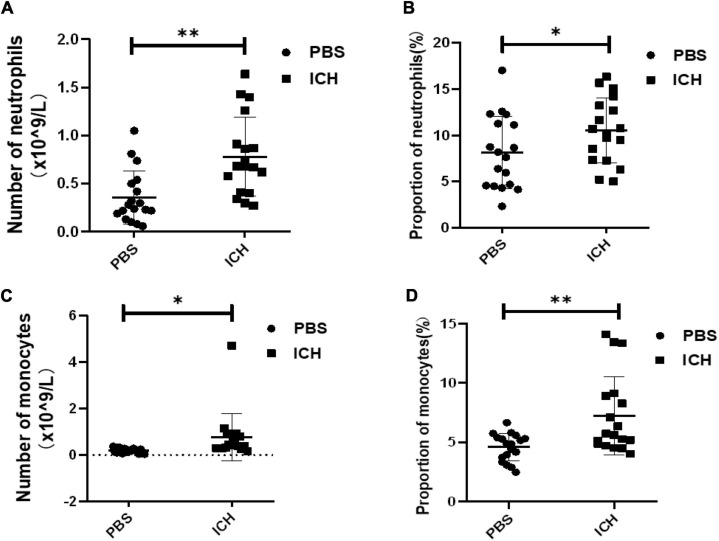
ICH can induce changes in the number and proportion of inflammatory cells in peripheral blood. **(A)** Quantity of neutrophils in peripheral blood (**P < 0.01, *n* = 18 in each group); **(B)** neutrophils/peripheral blood ratio (*P < 0.05, *n* = 18 in each group); **(C)** quantity of peripheral blood mononuclear cells (*P < 0.05, *n* = 18 in each group); **(D)** ratio of mononuclear cells to peripheral blood (**P < 0.01, *n* = 18 in each group).

Finally, we studied the changes in the number and proportion of neutrophils and monocytes in the peripheral blood of mice following ICH in order to evaluate the level of inflammation-related cells activated following ICH. Cytological examination of the peripheral blood of mice in each group demonstrated that neutrophils and macrophages increased in proportion and quantity following ICH (Figures 2A–D).

### Inhibition of myeloperoxidase activity reduced microglial proliferation following intracerebral hemorrhage

**FIGURE 3:**
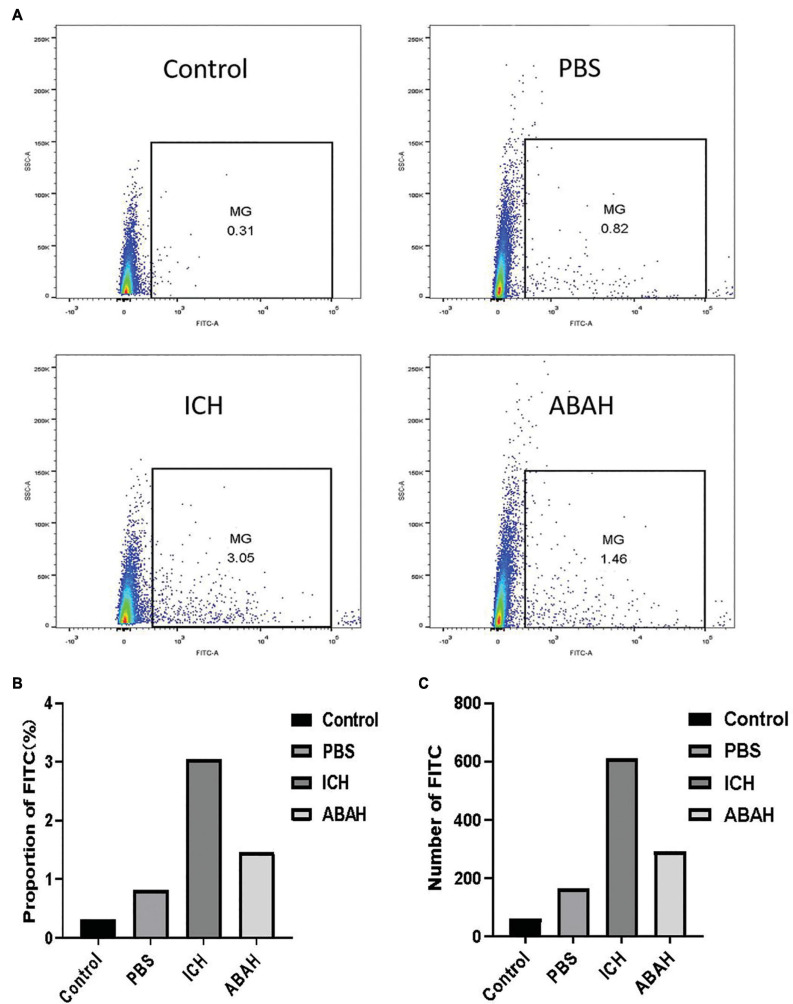
Inhibition of MPO activity reduces microglia proliferation following ICH. **(A)** Flow cytometry FITC channel, to detect the content of microglia in the control, PBS, ICH, and ABAH groups (set the total number of living cells to 20,000, *n* = 1 in each group); **(B)** proportion of FITC in the control, PBS, ICH, and ABAH groups; **(C)** number of FITC in the control, PBS, ICH, and ABAH groups.

In order to explore whether MPO activity affected changes in microglia following ICH, we used flow cytometry to count the microglia in the brains of mice 24 h after modeling. At the same time, in order to compare the levels of microglia in the normal physiological state, we specifically introduced a normal group as a control. The results showed that the number of microglia in the ICH group increased significantly, while those in the ABAH group decreased significantly (Figures 3A–C).

### Microglia and myeloperoxidase were activated following intracerebral hemorrhage

**FIGURE 4:**
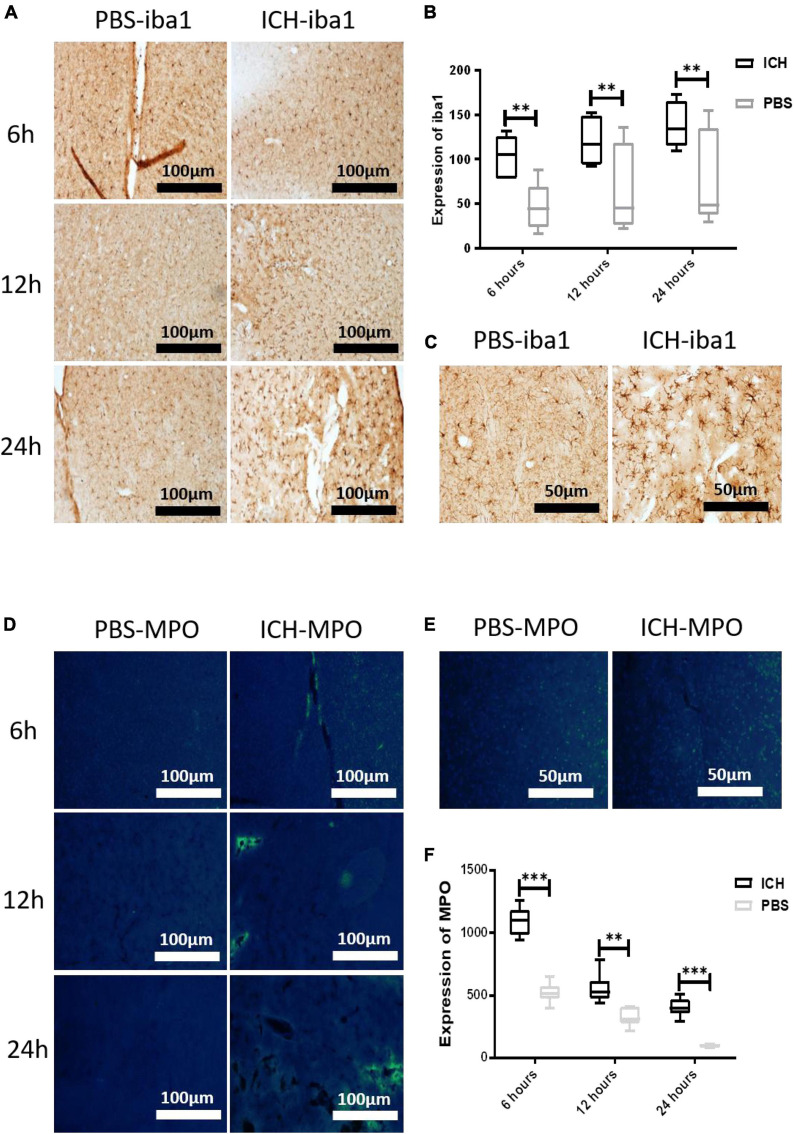
Microglia and MPO were activated following ICH. **(A)** Immunohistochemistry of the PBS and ICH groups at 6, 12, and 24 h post-injury. The labeled molecule was iba1 (10×, 20×); **(B)** statistics of iba1 positive expression (**P < 0.01, *n* = 6 in each group); **(C)** immunohistochemistry of iba1 in the PBS and ICH groups at 24 h post-injury (10×, 40×); **(D)** immunofluorescence of MPO in the PBS and ICH groups at 6, 12, and 24 h post-injury (10×, 20×); **(E)** immunohistochemistry of MPO in the PBS and ICH groups at 6 h post-injury (10×, 40×); **(F)** statistics of MPO molecular expression (**P < 0.01, ***P < 0.001, *n* = 6 in each group).

**FIGURE 5:**
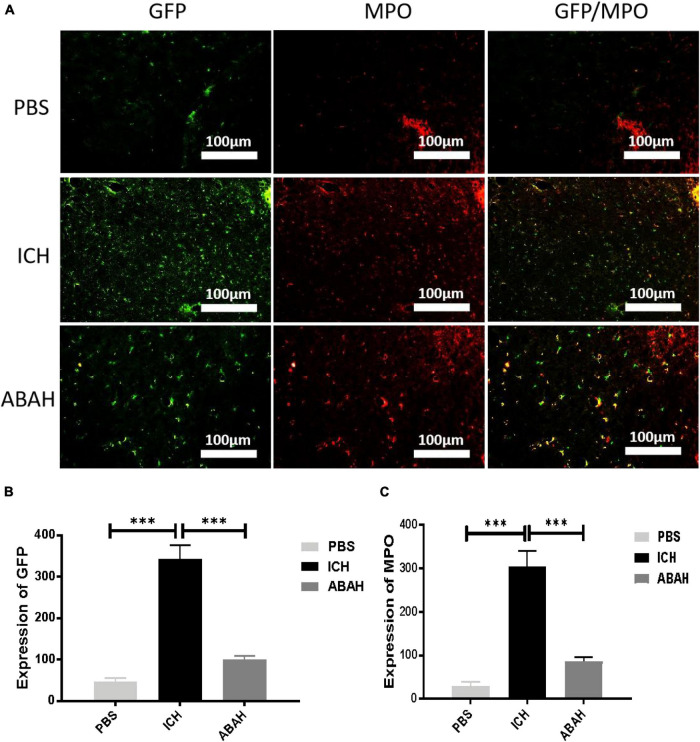
Inhibition of MPO activity reduces microglial proliferation following ICH. **(A)** Immunofluorescence double staining was performed in the PBS, ICH, and ABAH groups at 24 h post-injury. The labeled molecules were microglia and MPO; **(B)** microglia expression (***P < 0.001, *n* = 6 in each group); **(C)** MPO expression (***P < 0.001, *n* = 6 in each group).

Next, we used immunohistochemical and immunofluorescence techniques to detect microglia and MPO activity following ICH. In order to better understand the activation characteristics of microglia and MPO in the pathophysiology of ICH, we selected three time points (6, 12, and 24 h post-injury) in order to locate and quantify the expression of iba-1 and MPO in the mouse control group and the ICH group. The results showed that microglia were significantly activated in the ICH group compared with the PBS group, and the most significant activation was found at 24 h (Figures 4A–C). In the immunofluorescence detection of MPO, we found that the activity of MPO significantly increased following ICH, but the activation peak of MPO appeared at 6 h post-injury (Figures 4D–F). To further verify the relationship between microglia and MPO activation following ICH, we used a special CX3CR1 + /GFP gene-edited mouse, which could specifically display microglia via GFP fluorescence. Using immunofluorescence, we found that the expression of GFP and MPO in the ICH group was significantly increased compared with the other three groups, while the expression of GFP and MPO in the ABAH group was significantly reduced compared with the ICH group (Figures 5A–C). The results of the immunofluorescence determinations also showed that the expressions of MPO and GFP were spatially overlapped.

## Discussion

ICH is a serious central nervous system disease, accounting for approximately 10–15% of all stroke cases, and its mortality rate is as high as 30–67%. Therefore, ICH is one of the greatest challenges for current neurosurgical diagnosis and treatments (Li et al., 2020). Microglia are a special class of mononuclear phagocytes, which play a very important role in the development of the central nervous system and the occurrence, development, and prognosis of central nervous system diseases (Uff et al., 2022). Microglia, neutrophils, and macrophages in the blood are the main executors of inflammatory response to ICH, and inflammatory response is one of the main causes of poor prognosis for ICH patients (Liu et al., 2022).

Existing studies of therapeutic strategies involving microglia for ICH divide the latter into two main aspects: inhibiting the proinflammatory activity of microglia/macrophages and improving the regulatory properties of myeloid cells that display potential repair and anti-inflammatory properties. The former study subjects include Minocycline, complement 5a receptor antagonist (C5aRA), recombinant C1q/TNF-related protein 9 (rCTRP9), and the leukotriene B4 (LTB4) receptor antagonist, Bortezomib (a proteasome inhibitor), Fingolimod, etc. The study subjects of the latter aspect include Sphingosine-1-phosphate receptor (S1PR) agonists, statins, cannabinoid receptor-2 (CBR2) agonist, peroxisome proliferator-activated receptor γ (PPAR γ) activators, mammalian target of rapamycin (mTOR) inhibitors, Sinomenine, etc. Some of these medications and methods still lack the experimental verification necessary for their safe use in humans, but preclinical data support the use of inactivating agents or inhibitors of proinflammatory microglia/macrophages while enhancing the regulatory phenotype as part of a therapeutic approach to improving the prognosis of ICH (Wang, 2010; Shao et al., 2019; Bai et al., 2020).

Therefore, clarifying the molecular mechanisms related to the activation of microglia, neutrophils, and macrophages is the key to developing new therapies for ICH in the future. According to previous studies, MPO is an important regulatory molecule in the development of ICH, which can participate in the development and prognosis of stroke in various ways. Therefore, MPO is expected to become an important intervention target for ICH in the future (Wang et al., 2022). In summary, we have reason to believe that MPO can participate in the occurrence and development of ICH by changing the activation state of microglia. However, the relationship between MPO and microglial activation has not been experimentally verified in ICH, so this hypothesis was investigated in our study.

First, after the MPO inhibitor ABAH was given to ICH mice *in vivo*, the effects of MPO on ICH motor behavior were verified by behavioral experiments such as Longa score, open field test, and hind-limb clasping test. The experimental results showed that ICH could cause severe neurological dysfunction compared with the control group, which was highly consistent with the clinical manifestations of ICH patients. However, it was surprising that the MPO inhibitor ABAH improved motor behavior effectively following ICH. This preliminary experiment proved that the intervention of MPO activity is effective for the recovery of motor function following ICH, and it also verified our previous hypothesis that MPO can be used as a therapeutic target for ICH. In order to further explore the changes in MPO and microglia during the development of ICH, we analyzed these changes in the time dimension. The results showed that microglia were activated following ICH and peaked at 24 h. The expression of MPO also increased following ICH, peaking at 6 h following ICH. Because the peak of MPO expression appeared earlier than the peak of microglial activation, we believe that the high degree of expression of MPO is one of the causes of microglial activation following ICH. To further verify the relationship between microglia and MPO activation following ICH, we used a special CX3CR1 + /GFP gene-edited mouse, which could specifically display microglia *via* GFP fluorescence. Using the immunofluorescence detection of gene-edited mouse tissues, we found that the expression levels of GFP and MPO in the ICH group were significantly higher than those in the other three groups, while the expression levels of GFP and MPO in the ABAH group were significantly lower than those in the ICH group. At the same time, due to the consistency of GFP and MPO in terms of spatial expression, these experimental results further proved that MPO is closely related to the pathophysiological development of microglia following ICH. Therefore, in order to verify whether MPO can affect the activation of microglia following ICH, we used flow cytometry to count the number of microglia in normal mice, ICH mice, and mice treated with MPO inhibitor following ICH. The results showed that, compared with the normal physiological state, microglial cells proliferated significantly following ICH, but the inhibition of MPO activity could significantly inhibit this change. Therefore, based on the above experimental data, we proved that ICH induced proliferation of microglia, and this proliferation was closely related to MPO activity. At the same time, due to the destruction of the blood-brain barrier, neutrophils and macrophages in the blood entered the central nervous system following ICH and participated in the inflammatory response. Therefore, we performed peripheral blood cytology tests on the ICH mice, and the results showed that neutrophils and macrophages in the peripheral blood of ICH mice increased in both proportion and quantity.

Our study, however, had certain limitations. We did not validate the specific type of activated microglia in our experiments, and we will continue to supplement our data with this information in subsequent experiments. In addition, we did not verify the regulatory role of MPO activity in microglia proliferation using *in vitro* experiments, nor did we discuss the signal transduction mechanism in the activation process. We plan to further clarify the specific activation mechanism between MPO and microglia by means of molecular and cellular experiments, clarify the interaction between MPO and microglia, and find other possible targets for additional MPO inhibitors.

In summary, our studies have shown that ICH can cause a large degree of proliferation of microglia in the brain and increase the number of neutrophils and macrophages in the circulation. The increased MPO activity following ICH is closely related to the proliferation of microglia, and so is expected to become an important therapeutic target for ICH in the future.

## Data availability statement

The original contributions presented in this study are included in the article/supplementary material, further inquiries can be directed to the corresponding author.

## Ethics statement

This animal study was reviewed and approved by the Medical Laboratory Animal Ethics Committee of Lanzhou University Second Hospital.

## Author contributions

WZ, YW, JS, and YZ contributed to the conception and design of the study. WZ and YW performed data analysis and drafted the manuscript. YW and YZ participated in editing the manuscript. JS has made contributions to the implementation of some topics. All authors contributed to the article and approved the submitted version.

## Footnotes

1
https://www.flowjo.com

2
https://www.graphpad.com
